# “Sexual harassment definitely involves a hierarchical transgression”: Key informants´ discourses on sexual harassment in European universities

**DOI:** 10.1371/journal.pone.0355556

**Published:** 2026-08-12

**Authors:** Esther Ríos-Albert, Ariadna Cerdán-Torregrosa, Eva Espinar-Ruiz, Sylwia Jaskulska, Barbara Jankowiak, Lidia Del Piccolo, Valeria Donisi, Francesca Dal Maso, Sofie Avery, Mafalda Sousa, Estefânia Silva, Sofia Neves, Viktoria Stifter, Marlies Wallner, Carmen Vives-Cases

**Affiliations:** 1 Department of Community Nursing, Preventive Medicine and Public Health and History of Science, University of Alicante, Alicante, Spain; 2 Department of Sociology II, University of Alicante, Alicante, Spain; 3 Faculty of Educational Studies, Adam Mickiewicz University, Poznań, Poland; 4 Department of Neuroscience, Biomedicine, and Movement Sciences, University of Verona, Verona, Italy; 5 Department of Philosophy, University of Antwerp, Antwerp, Belgium; 6 Department of Philosophy and Moral Sciences, Ghent University, Ghent, Belgium; 7 Department of Social and Behavioral Sciences, University of Maia, Maia, Portugal; 8 Interdisciplinary Center for Gender Studies (CIEG/ISCSP-ULisboa), Lisboa, Portugal; 9 Department for Health and Social Sciences, University of Applied Sciences Burgenland, Eisenstadt and Pinkafeld, Eisenstadt, Austria; 10 CIBER of Epidemiology and Public Health (CIBERESP), Madrid, Spain; Lamar University, UNITED STATES OF AMERICA

## Abstract

Understanding how university members conceptualize sexual harassment is crucial, as their perceptions influence recognition, reporting, and response to the issue. The aim of this study was to explore the discourses of key university informants about sexual harassment within universities, including student representatives, university staff, and institutional representatives in equality and diversity. A qualitative approach using semi-structured interviews (November 2023 to April 2024) with 90 key informants from six universities located in Austria, Belgium, Italy, Poland, Portugal, and Spain. Data were analyzed using Critical Discourse Analysis. Results show that sexual harassment is perceived as a multidimensional phenomenon encompassing physical, verbal, non-verbal, offline, and online behaviors. Participants’ accounts consistently emphasized the transgression of interpersonal boundaries, the absence of consent, power imbalances, and negative consequences on victims-survivors’ well-being. Participants’ definitions fell along a continuum of three discursive approaches— *normative*, *experience-based*, and *integrative*—which varied according to gender and institutional role. Regarding potential victim-survivors, two discourses emerged: an *intersectional discourse* frames vulnerability as the result of overlapping social inequalities—such as gender, age, hierarchical position, sexual orientation, ethnicity, migration background, disability, and/or economic dependence; and a *situational discourse* emphasizing risks associated with structural dynamics of academia, including hierarchy and dependency, regardless of individual characteristics. Concerning potential perpetrators, a *hierarchical discourse* was dominant among participants, alluding to men in positions of authority and leadership within the university. Two minoritarian and alternative discourses emerged: a *peer perpetration discourse*, situating harassment among individuals of similar hierarchical status, particularly students; and a *psychological characteristics discourse*, explaining perpetration in terms of individual traits, such as narcissism, manipulation, or egocentrism. Our findings suggest the need for university policies that integrate intersectional and gender-transformative perspectives and promote egalitarian and safer academic environments.

## Introduction

Sexual harassment (SH) is a global challenge, causing severe consequences in victims-survivors’ lives [1]. The Istanbul Convention [2] defines SH as “any form of unwanted verbal, non-verbal or physical conduct of a sexual nature with the purpose or effect of violating the dignity of a person, in particular when creating an intimidating, hostile, degrading, humiliating or offensive environment” [Article 40, p. 11]. SH might be perpetrated in-person or online [3]. However, as the concept encompasses diverse terms and reflects historical ambiguity regarding its boundaries, SH can be understood from both subjective and objective approaches [4–6]. The subjective approach refers to idiosyncratic individual perceptions based on victims-survivors’ experiences and perpetrators’ intent [5–9], while the objective approach relies on legal criteria to determine whether a behavior qualifies as SH. i.e., as a crime [6,10]. The first one has been criticized due to its potential biased nature, that can lead to justify the perpetrator’s actions and shift the responsibility onto the victims [6,10]. Conversely, despite SH being formally typified in the Law, the objective approach reveals difficulties in operationalizing certain elements of the concept, for example, defining a hostile environment can be challenging [11]. Consequently, ‘gray areas’ emerge, reflecting the conceptual ambiguity surrounding SH. These include subtle behaviors, such as sexist jokes, insistent looks or inappropriate comments, although experienced as transgressive and unwanted by victims, are often not recognized as SH [7,12]. Furthermore, the massive use of information and communication technologies has increased online SH, adding complementary challenges for its detection and regulation [13].

Scientific research on social perceptions of SH is extensive. Perceptions can be defined as a complex construct influenced by individual characteristics, personal experiences, political and cultural background, and social mechanisms [14]. The labeling of behaviors as SH and the meanings attributed to them depend on overlapping dimensions such as individual sensitivity, interpersonal boundaries, and power dynamics. Thus, following the socio-ecological model approach, perception is not formed in isolation but is shaped by multiple interacting factors at the individual, interpersonal, and community levels [15,16]. At the individual level, factors such as gender, age, ethnicity, social class, sexual orientation, and experiencing or witnessing SH play a key role in shaping the understanding of SH or another form of gender-based violence [5,17–20]. At the interpersonal level, factors such as peer norms can influence attitudes toward SH [18]. At the community level, equality policies and legal sanctions against perpetrators have a positive influence on attitudes towards SH and other forms of gender-based violence, by increasing awareness, reducing victim-blaming, and reinforcing community support for accountability measures [18,21,22]. Moreover, factors such as gender stereotypes in mass media and exposure to violent pornography, particularly among young men, are associated with negative attitudes toward women and an increased likelihood of SH perpetration [18,23]. Finally, SH has been normalized in many contexts, making it difficult for victims to identify themselves as such [22,24]. Those factors are dynamic and are associated with both victims’ and perpetrators’ attitudes, behaviors, and emotions towards SH [25].

Understanding how individuals conceptualize SH and other forms of gender-based violence is crucial, as their perceptions can affect their ability to recognize violent behaviours, their willingness to report incidents, the strategies they adopt to prevent them, and the actions they take to seek for help [18,26–28]. In universities, sexist structures, gender inequalities, and discriminatory practices persist, enabling SH and other gender-based violence due to historically unequal power relations based on gender, age, status, and class [29]. Studies in the field concluded that SH disproportionately affects women, members of the LGBTQIA+ community, young people, those who reported a disability or chronic illness, and people who belonged to a minority ethnic group [30–32]. A recent worldwide review [33] showed that the overall prevalence of SH among students stands at 36.9%, with rates of 43.7% for women, 16.8% for men, and 33.4% for non-binary people. According to the European UniSAFE survey [31], almost one in three students and staff (31%) state having experienced SH within their institution.

Although limited, previous research has explored how the university’s community members, including students and staff, define, identify, classify, and perceive SH behaviors [18,19,34–36]. These studies suggest that perceptions of SH are frequently influenced by gender, age, and position within the university. For instance, women, especially staff, tend to recognize a wide variety of physical, verbal, and psychological forms of SH, including more subtle ones such as inappropriate comments, lascivious looks, or excessive physical proximity [19,34,35,37]. In the same sense, women perceive a greater occurrence of SH when compared to men [38]. In contrast, men, especially students, tend to minimize SH, recognizing the more extreme forms, such as sexual coercion or explicit sexual advances [19,37]. This gender difference in identifying SH reflects its perception as a form of violence that disproportionately affects women, who—because they are more likely to have experienced it—tend to be more sensitive to a broader range of behaviors [22,35,39]. In this context, members of the university community tend to perceive men, especially professors, as potential perpetrators who may take advantage of their superior institutional status [35,37,39].

Recent political and social initiatives, like the #MeToo movement, point to the need to explore how ongoing transformations shape current perceptions and responses to SH within the university community [40]. As most previous research has focused mainly on the perceptions of students and university staff, it is also necessary to capture the views of other actors involved in understanding and responding to SH in the university environment. In particular, professionals responsible for managing cases and implementing institutional protocols—such as psychologists, lawyers, equality technicians, sociologists, and social workers—include both university-based staff and external professionals collaborating with institutional services (e.g., from NGOs, local councils, or other public institutions) working on equality issues related to the university context. These actors play a key role in the practical application of normative frameworks, the support of victims and survivors, the implementation of preventive measures including institutional campaigns and training, and the development of equality policies. Therefore, considering these institutional representatives as key informants allows for a more comprehensive analysis of how they conceptualize and interpret SH, which could provide valuable insights for the development of more effective and protective policies, measures, and strategies to enhance specialized support services [41,42].

The present study explores the discourses of key university informants about SH within universities, including student representatives, university staff, and institutional representatives in equality and diversity.

## Materials and methods

### Study design

This study is part of the European project “Uni4Equity” (2023–2026) based on inter-institutional cooperation and conducted at the University of Alicante (UA, Spain), University of Verona (UNIVR, Italy), University of Maia (UMAIA, Portugal), University of Antwerp (UAntwerp, Belgium), Adam Mickiewicz University in Poznan (AMU, Poland), University of Applied Sciences Burgenland (UASB, Austria), Aplica Research and Transfer S. Coop. Mad (Spain) and CESIE ETS (Italy). The main objective of the project is to strengthen universities’ capacities to identify, map, and respond to online and offline SH in the workplace and other relevant environments (classrooms, digital space), with an explicit (but not exclusive) focus on social minority groups [43]. This collaborative perspective contributes to a comprehensive understanding of the issue, by considering the variety of university contexts.

Due to the exploratory nature of this study, a qualitative methodology was applied with data from semi-structured interviews. This technique enabled participants to provide detailed and personal accounts in a confidential environment.

### Ethical considerations

This study adheres to all the ethical principles of scientific research with human participants, namely confidentiality, anonymity, and safety, and complies with the European Union’s General Data Protection Regulation. It was approved by the Ethical Committee of the UA (UA-2023-03-27); the UNIVR (n. 24/2023); the UMaia (N. º 151/2023, July 25th, 2023), the UAntwerp (SHW_2023_38_1, 20 November 2023); the AMU (N. º 19/2022/2023); the UASB (06/18/2023).

### Sample, recruitment, and data collection

The study employed a sample triangulation strategy based on the inclusion of three key informants to capture the diversity and complexity of perceptions surrounding SH in universities. The sample comprised 1) student representatives, 2) university staff, and 3) institutional representatives in equality and diversity from different universities. This last interviewees group combines both internal and external professionals involved in university’s broader social and institutional context. Specifically, it included internal professionals (n = 25), such as staff working in university equality and diversity offices and staff involved in SH resources or administrative offices (e.g., psychologists, lawyers, equality officers, sociologists, and social workers). It also included external professionals collaborating with university services (n = 11), such as members of NGOs, local councils, and other public institutions working on equality and youth-related issues within the university context across the participating countries. This triangulation of perspectives enabled the comparison and integration of discourses across these three groups, enriching the analysis by revealing how different key actors interpret, experience, and operationalize SH, and by highlighting possible similarities or contradictions between discourses, institutional policy, and practice.

The sample was recruited considering the context of each university, to ensure diversity and plurality of information. Purposive sampling was used across participating universities, following the criteria of gender, occupational and academic profile, and membership in diverse organizations such as the LGBTQIA+ community, ethnic minority groups, and people with disabilities. As a complementary recruitment strategy, the snowballing technique was when it was difficult to access key informants, thus ensuring a variety of perspectives. All participants were informed about the study goals, the methodology involved, the voluntary nature of their participation, data protection, and the right to withdraw at any time without consequences. Informed consent was signed before the interview was conducted, and authorization for audio recording was requested.

A total of 90 semi-structured and individual interviews with 68 women, 21 men, and 1 non-binary key informants were conducted, in-person (n = 60) or online (n = 30), by researchers, research assistants, and staff from the project, all with training and experience in managing SH cases. Participants’ ages ranged from 20 to 76, with an average of 43.7 years. The sample comprises 23 student representatives, 31 university staff, and 36 institutional representatives (Table 1). Interviews were conducted in the vernacular language at each site (Spanish, Italian, Portuguese, Dutch, Polish, and German), audio-recorded and transcribed for analysis.

**Table 1:** Sociodemographic Characteristics.

	Total number of interviews	Format	Average age of participants	Sex/ gender	Profiles of the participants
Site 1: UA	**16**	**In person:10** **Online:6**	**42,9** **Youngest = 21** **Oldest = 48**	**Women: 16**	**University Staff: 5** **Student representatives: 5** **Institutional representatives: 6**
Site 2: UNIVR	**15**	**In person: 9** **Online: 6**	**44,6** **Youngest = 21** **Oldest = 73**	**Women: 9** **Men: 6**	**University Staff: 4** **Student representatives: 4** **Institutional representatives: 6**
Site 3: UMaia	**13**	**In person:5** **Online:8**	**44,4** **Youngest = 20** **Oldest = 76**	**Women: 9** **Men: 4**	**University Staff: 5** **Student representatives: 3** **Institutional representatives: 5**
Site 4: AMU	**16**	**In person: 10** **Online: 6**	**42,6** **Youngest = 21** **Oldest = 64**	**Women: 12** **Men: 4**	**University Staff: 5** **Student representatives: 4** **Institutional representatives: 7**
Site 5: UASB	**15**	**In person: 11** **Online: 4**	**44** **Youngest = 22** **Oldest = 60**	**Women: 11** **Men: 4**	**University Staff: 5** **Student representatives: 4** **Institutional representatives: 6**
Site 6: UAntwerp	**15**	**In person: 15**	**<30:** **6** **30 to 50: 1** **>50: 8**	**Women: 11** **Men: 3** **Non-binary:1**	**University Staff: 6** **Student representatives: 3** **Institutional representatives:6**
Total sample	**90**	**In person: 60** **Online: 30**	**43,7**	**Women: 68** **Men: 21** **Non-binary: 1**	**University Staff: 31** **Student representative: 23** **Institutional representatives:36**

The interviewers followed an initial and common script, developed in English within the Uni4Equity project, which structured the interviews into several thematic sections: (1) perceptions of SH; (2) knowledge of available resources; (3) perceptions and assessment of those resources; (4) barriers and facilitators in accessing resources; (5) perceived risks, opportunities, and policy recommendations; (6) other relevant issues not previously addressed. Given the semi-structured nature of the interviews, each research team adapted the script to their local context by incorporating specific examples and terminology while maintaining the core thematic structure to ensure comparability across sites. For the present study, the analysis focused primarily on the section titled “Perceptions of SH,” which included the participants’ understandings and definitions of SH, the situations they related to this issue, and their perceptions towards victims-survivors and perpetrators. This section was further supported by a cross-sectional reading of the full interview transcripts. The average length of interviews was 1 hour and 02 minutes. This study is cross-sectional, as all interviews were conducted during a single data collection period, between 3 November 2023 to 18 April 2024. Recruitment did not begin simultaneously across study sites. Each participating university conducted data collection during specific periods within this overall timeframe. Detailed start and end dates for recruitment at each site are provided in Table 2.

**Table 2:** Recruitment periods for each participating university.

Participating universities	Recruitment start date	Recruitment end date
University of Alicante	03/11/2023	06/02/2024
University of Verona	13/12/2023	15/02/2024
University of Maia	24/11/2023	23/02/2024
Adam Mickiewicz University	22/11/2023	5/02/2024
University of Applied Sciences Burgenland	14/11/2023	20/12/2023
University of Antwerp	18/03/2024	18/04/2024

### Data analysis

A Critical Discourse Analysis was conducted using the qualitative analysis software Atlas.ti.24. Following van Dijk´s approach [44,45], discourse is understood as a multi-dimensional phenomenon in which social cognition and context interact, with everyday language reflecting how ideologies influence personal and social perceptions of reality. In this framework, discourse is not merely a sequence of linguistic structures, but is rooted in processes of meaning production and situated use within specific social contexts. Critical Discourse Analysis aims to identify the social structures of discourse and how they shape and reproduce social power dynamics. This perspective goes beyond what is explicitly said, exploring the underlying meanings, contradictions, and assumptions in participants’ accounts. In this study, Critical Discourse Analysis was used to understand how key informants’ perceptions at the universities are influenced by dominant discourses, power dynamics, and social representations of SH.

Individual interviews were the core material for the analysis. Firstly, a deductive coding process was carried out to identify patterns in meaning-making across interviews. Research teams used a structured and common template to record the application of each code and to provide textual support. This initial coding phase was complemented by repeated familiarization with the entire data corpus and a review of the relevant literature, which together informed the interpretative process. Through this iterative analysis, three discursive categories were constructed (conceptualization of SH; representations about SH victims-survivors; and representations about SH perpetrators), which served as the analytical framework for exploring SH. Discursive categories are understood as analytically constructed groupings derived from the coding process that organize participants’ discourses or narratives. All of this allowed for the identification of dominant discourses, understood as those with greater presence across participants’ narratives, as well as alternative discourses, which referred to those with lesser presence that expressed different interpretations among the participants.

Subject positions were also highlighted as part of the analysis [46], which refers to the identities or roles individuals adopt in discourse, shaped by their social context and personal experiences. This approach enabled the identification of how university position (staff, student or institutional representatives) and gender shaped participants’ discourses and interpretations of SH. Moreover, ambivalences were also identified [47], which refers to the multiplicity of representations, beliefs, and attitudes towards social phenomena expressed by participants. Ambivalence is understood as a dynamic and context-dependent feature of meaning-making that emerges in the interaction between individual and social dimensions. Therefore, it is an action that is not necessarily contradictory, but rather it is experienced as multiple and dynamic.

While the first author led the analytical process, coauthors from the diverse research teams participated in a continuous and iterative process of categorization, discussion, and interpretation, aiming to reach inter-coder consensus. Codes were regularly compared and discussed across researchers to identify similarities, relationships, and potential divergences in interpretation. When disagreements arose, these were addressed through collective discussion and re-examination of the quotes and their contextual meaning within the full data corpus.

## Results

Key informants’ discourses regarding SH in the university context were organized into three discursive categories: (1) Conceptualization of SH which explores participants’ perceptions of this issue and its main roots; (2) Representations of SH victims-survivors which explores participant´s discourses on the profiles and vulnerabilities of SH victim-survivors; (3) Representations of SH perpetrators, which focuses on participants’ discourses about SH perpetrators. These discursive categories, which are not mutually exclusive, are observed below, and several differences were evident according to participants’ university position and gender.

1. **Conceptualization of SH**

The participants, regardless of their university role or position, identified under the term SH a wide typology of inappropriate behaviors, both direct and indirect, including physical, psychological, verbal, non-verbal, and online manifestations. In their narratives, they highlighted specific examples such as unwanted comments, looks or gestures with sexual connotations, non-consensual sending of sexual messages or images, unwanted touching, opinions about physical appearance, unsolicited compliments, and jokes with sexual content. No gender differences were observed, as both women, men, and non-binary participants recognized this diversity of behaviors.

*“I’d see it more as a behavior that can be verbal, indirect, physical, or something else.”* (UNIVR_05, SR, man)

*“I think it also includes verbal and non-verbal actions. It can involve, for example, joking or commenting on someone’s clothing or behavior.”* (AMU_05, US man)

*“It ranges from just a look to a sexist joke. […] In schools, sexist jokes tend to belittle women and confine them to a stereotypical role.”* (UNIVR_15, IR, man)

*“From professors who make remarks about the way they dress or about their neckline.”* (UA_11, US, woman)

Online SH was explicitly mentioned and described as a homonymous concept to general SH, but is specifically situated in the digital context (e.g., social media, virtual campus, or institutional channels). However, participants cautioned that online SH carries additional risks and impacts compared to offline SH. The anonymity of online environments was considered a factor that can encourage, disinhibit, and facilitate perpetration, while hindering perpetrator accountability. Another factor is a victim´s feeling of constant control, because the perpetrators may contact them repeatedly through calls or messages outside of work or school hours, showing greater persistence and intrusion into their private lives.

*“For me, online sexual harassment is the same way it can happen in person. It’s just that you’re using social media. You’re using either WhatsApp or, as we’ve had in some cases, the university’s own channels.”* (UA_02, IR, woman)

*“Online, you can actually have more anonymity. That encourages this type of violence to occur, so maybe more in this area.”* (AMU_04, SR, woman)

*“The big concern is social media... People put on another skin, they become another person, so they’re avatars. I think that’s the big risk. It’s the big challenge. Maybe it’s still much more camouflaged, because it’s not visible, is it?”* (UMAIA_07, US, man)

*“I think that’s a mechanism happening online: the face disappears, we all become objects, and feel more entitled to use social norms that would inhibit us face-to-face. We’re more likely to treat the other as always available.”* (UNIVR_15, IR, man)

*“Technology is a new tool for exercising control 24 hours a day in a permanent and constant way. I think it’s very dangerous because of social media.”* (UA_13, IR, woman)

Across participants’ accounts, a shared conceptualization of SH emerged around common behavioral elements. First, the transgression of interpersonal boundaries related to sexual aspects. They defined SH as any transgressive behavior (physical, verbal, or online) that invades a person’s privacy, such as physical approaches, unwanted touching, intrusive gestures or comments, messages, etc. Second, the absence of consent and the notion of “unwanted” emerged as central elements, including any interaction with sexual connotations that occurs without the explicit consent of the recipient. Third, power imbalance emerged as a key factor in the understanding of SH in the university context. Participants, mainly staff and institutional representatives, mentioned that hierarchical structures based on gender, age, and academic or job position facilitate SH by enabling abuses of authority, in which people in higher positions can take advantage of their status to exert control over others in lower positions (e.g., coercion or blackmail to obtain sexual favors). They perceived that these traditional and unequal dynamics encourage impunity for perpetrators and silence victims, who often feel powerless due to their academic, professional, and economic dependence. Finally, female participants -across including students, staff, and institutional representatives- emphasized the negative impact of SH on the physical, emotional, social, academic, and occupational well-being of those who experience it, highlighting that this impact is rooted in the victim’s perception of discomfort and harm.

*“I understand it as a violation of someone’s personal space, crossing boundaries, something with a sexual basis or something that may be interpreted as such by the victim. For example, touching, but also intrusive gestures or even verbal behavior.”* (AMU_03, SR, woman)

*“Sexual harassment can be defined as an act directed at a person without their consent.”* (UNIVR_06, IR, woman)

*“And of course, touching you when you don’t want that. Something along those lines, or even when it happens in writing, through words.”* (UASB_02, US, woman)

*“Behaviours of a sexual nature that are annoying, that don’t have the consent of the other person, and that make you feel uncomfortable.”* (UA_14, SR, woman)

*“Sexual harassment definitely involves a hierarchical transgression.”* (AMU_06, IR, woman)

*“The harassers have a role, let’s say, at a professional level, they hold a hierarchically superior position, right? And they also use a kind of blackmail, like: ‘you depend on me, meaning that if I make a decision, you could be pushed out or your professional career might not continue’.”* (UA_10, US, woman)

*“But the moment that behavior impacts that person’s well-being, how that person feels here within the organization”* (UAntwerp_14, US, woman)

A recurring theme in the interviews was the normalization and tolerance of subtle behaviors that, despite being perceived as inappropriate, are often not explicitly recognized as SH. Staff and institutional representatives, regardless of their gender, drawing on their personal experiences as lecturers, bystanders, or professionals, reported that certain behaviors, such as persistent attention, public remarks, or jokes, are frequently tolerated or dismissed by the university community. Both profiles noted that the subtlety and ambiguity of these behaviors create “gray areas” in which cultural legacies and social norms blur the lines of acceptability, fostering a climate in which inappropriate actions can be downplayed, misinterpreted, or labeled as “inappropriate insistence” or “harmless flirting”.

*“And I do think there are certain behaviors of which hopefully we would all say no, this is just unacceptable.” That gray area is obviously where it’s tricky.”* (UAntwerp_14, US, woman)

*“The problem with harassment is that it’s, it’s, I believe, very difficult to draw the line — the red line.”* (UMAIA_02, IR, man)

*“I’ve witnessed behaviors from male professors toward female colleagues that, while not outright harassment, I consider inappropriate—like making flattering remarks to a woman during a public meeting, or being overly persistent in trying to interact with her. […] I wouldn’t call it harassment, but rather inappropriate insistence, especially because it happens publicly.”* (UNIVR_13, US, man)

Although participants, regardless of their university position, expressed a common definition of SH, they adopted different positions towards this issue. Beyond this shared understanding, three distinct discourses were articulated through which participants perceived SH in the university context depending on their institutional profiles: normative, experience-based, and integrative.

***Normative discourse.*** On the one hand, a normative perspective emerged, in which SH is framed within legal concepts (e.g., sexual freedom or personal dignity) and institutional definitions, such as those established in law, protocols, codes of conduct, and lists of standardized behaviors. Women and non-binary staff members were more likely to adopt the normative discourse when discussing experiences of SH. From this subject position, participants advocated for regulatory frameworks that address the diversity of individual sensitivities and interpretations of SH. In this regard, they emphasized external validation—through legal definitions in institutional protocols and codes of conduct—as a mechanism that provides clarity and consistency essential for identifying and classifying SH.

*“Is a way of affecting sexual freedom, which is a personal freedom […] And for me it is so deeply rooted in one’s own personality and personal dignity that I would almost say it is the most serious attack on freedom.”* (S1_US_12, woman)

*“Okay, for sexual transgressive behavior, myself I just follow the law here in our research.”* (UAntwerp_10, US, woman)

*“I think that can also be dangerous to call something purely personal and subjective. You also have to have the courage as a society to recognize it as transgressive before you also start experiencing it personally as transgressive.”* (Uantwerp_05, US, non-binary)

***Experience-based discourse.*** On the other hand, the experience-based perspective emphasized the importance of victims-survivors´ interpretations, experiences, and feelings of discomfort in identifying behaviors as inappropriate. The students’ discourses, mainly women, tended to align with this position, as they drew on their own experiences, both as victims and bystanders, to define SH. In these examples, female students interviewed mentioned subtle or less visible forms of SH, such as unwanted comments, sexual jokes, sexually explicit song lyrics, or suggestive behavior. They noted that these behaviors tended to evoke feelings of discomfort and stress, which they considered essential for recognizing and labeling the actions as SH.

*“For me, whether something is harassment also depends on how it’s perceived: if it makes someone uncomfortable, then it’s harassment.” (UNIVR_04, SR, woman)*

*“The most basic example would be someone singing a song with sexual content directly to me, making me feel uncomfortable.” (UMAIA_06, SR, woman)*

*“And during an event where we had a booth, he [a male lecturer] looked me up and down, really scanned me, and made a comment about my clothing and how it looked. In principle, nothing terrible, but the feeling was unpleasant”* (UASB_SR,03, woman)

***Integrative discourse.*** This position was mainly adopted by male and female institutional representatives, reflecting an integrative perspective that seeks to balance normative criteria with an understanding based on the experience of victims. In this sense, participants, regardless of their gender, classified SH as a “crime” punishable by law, thus offering a definition aligned with regulatory and institutional criteria (e.g., protocols, codes of conduct, behavioral lists). Therefore, drawing on their professional experience managing SH cases, institutional representatives referred to the law as a framework for objectivity and clarity in the management of SH cases, allowing the university community (whether as potential victims, bystanders, and/or professionals involved) to identify certain behaviors as SH, without relying only on individual perception. However, they also mentioned that legal frameworks do not always capture the full complexity of SH. For this reason, they also refer to the experience-based dimension, emphasizing that SH is defined above all by the victim-survivor’s perception of the behavior as unwanted and transgressive. Consequently, again guided by their own professional experience, the case-by-case approach emerged as a way to address each SH situation individually, taking into account normative criteria but also the recipient’s story and the specific context in which it occurs.

*“And here, we fundamentally have the Equal Treatment Act, which on the one hand defines what constitutes sexual discrimination (...).”* (UASB_01, IR, woman)

*“<<Unwanted>> is key, because it places the responsibility of defining the behavior on the recipient. It’s the recipient who decides.”* (UNIVR_01, IR, woman)

*“SH is defined based on the perspective/experience of the affected individuals, regardless of the labor or criminal law framework.”* (UASB_03, IR, man)

*“Actually, you’re really in case-by-case work there. [...] When I look at the cases I was confronted with, each case is very special and very particular with its own surprising things you never thought of.”* (UAntwerp_01, IR, man)

1. **Representations about SH victims-survivors**

Within this discursive category, two different discourses emerged regarding the potential profile of a victim of SH in the university context: an *intersectional discourse* and a *situational discourse*. The *intersectional discourse*, which emerged as a dominant perception, characterized the potential profile of SH victim in the university context as a person in a situation of social vulnerability due to multiple and intersecting social determinants, such as gender, sexual orientation, ethnicity, migration background, etc. Interviewees understood vulnerability not as a single factor but as the product of overlapping social structures of inequality that shape individuals’ exposure to SH. In contrast, the *situational discourse*, which emerged as an alternative perception, emphasized that SH can potentially affect any member of the university community, regardless of gender or social background.

*“These things must be seen through this new big word, increasingly adopted, which is intersectionality, right? Each person is a person, with all their baggage, with everything they bring.”* (UMAIA_05, IR, man)

***Intersectional discourse.*** Gender emerged as a key determinant in shaping SH victimization. This common perception across three interviewee profiles identified women as the primary SH victims-survivors and recognized SH as a form of gender-based violence. Especially, participants mentioned that female students (e.g., degree, PhD) or females in the early stages of their professional careers (e.g., part-time lecturers, junior researchers, residents, etc.) are particularly vulnerable due to their position of subordination to figures with higher hierarchical power (e.g., male lecturers, thesis director or supervisor). Staff, student and institutional representatives, particularly women, mentioned financial dependence on the perpetrators (through scholarships, fellowships, employment contracts, etc.) as the main barrier to speaking up and seeking help.

*“I think it is that which we suffer, well.... I would say women, by the fact of being women.”* (UA_SR_06, woman)

*“Harassment issues are seen as a gender issue, a gender violence issue, because it is mostly women who are victims of this crime.”* (UMAIA_05, IR, man)

*“Female PhD students and residents in particular are often afraid to speak up, because it could damage their career.”* (UNIVR_04, SR, woman)

*“It is more difficult to evade from SH when being hierarchically on a lower level”* (UASB_04, IR, man)

While gender emerged as a central axis in participants’ accounts of vulnerability, other social factors are mentioned, especially among female participants, including sexual orientation, gender identity, ethnicity, migration background, lack of social support, having a disability, having suffered previous experiences of violence (e.g., bullying), or an economically disadvantaged situation. These factors were described as shaping the social position of victims and limiting their ability to report or seek help. For example, participants noted that migrant people often face racial and cultural stereotypes, which intersect with structural barriers such as language, lack of social network, or unfamiliarity with the institutional context. Similarly, the LGBTQ+ community and gender-nonconforming people (e.g., non-binary people and people who do not follow gender stereotypes) were mentioned as potentially vulnerable.

*“I mean, what comes to my mind, I guess also because it’s what catches my attention, is the way in which vulnerable social circumstances intersect.”* (UA_02, IR, woman)

*“You are even more vulnerable when you have a sexual orientation that may not conform to the norm, and I think that makes you even more vulnerable.”* (UASB_02, IR, woman)

*“If you are a person with no schooling, in a context of greater vulnerability, let’s imagine black and roman [...] maybe your probability of suffering harassment is higher.”* (UMAIA_05, IR, man)

*“A person with a physical disability can also experience sexual harassment. We know that this actually happens quite frequently.”* (UASB_01, IR, woman)

*“International students don’t all have the same values, norms, or customs in their culture. So yes, sometimes a hug from someone can suddenly be very overwhelming for someone who is not used to it at all, but for others it is really the norm in their country of origin.”* (UAntwerp_03, SR, woman)

*“Foreigners who are uprooted [...] have a more disadvantaged socio-economic situation, I think they are more vulnerable, because they don’t have anyone to protect them or speak up for them. They’re alone”* (UMAIA_04, IR, woman)

*“Although it might seem that mostly women are affected, in my opinion, non-cisgender people are currently the most at risk, because first of all, it’s very difficult for them to even report to institutions – especially universities. In public perception, a university is a rigid structure that doesn’t accept difference”* (AMU_02, SR, man)

*“I also think of boys perceived as effeminate or not fitting the traditional, stereotypical image of masculinity—whether they’re gay or not.”* (UNIVR_13, US, man)

***Situational discourse.*** Rather than focusing on a specific profile of SH victims defined by individual or social characteristics, this alternative discourse, present across different interviewee profiles, emphasized the context in which SH occurs, particularly the higher education system. It framed SH as a situational phenomenon, pointing to negative characteristics of the university environment, such as competitiveness or hierarchical organization, that may facilitate SH victimization.

*“It’s hard to generalize and name a group. I would rather frame it as a matter of situation – it’s situational. And I think that at the university, in a threatening situation, essentially anyone who is in a dependent position by definition can be at risk.”* (AMU_IR_03 woman)

Within this framework, a subject position emerged among male and female student representatives that suggests a gender-neutral perspective, emphasizing that “anyone” within the university community-regardless of gender or another social factor- can experience SH. From this discourse, the risk of experiencing SH is understood in relation to the unequal power positions and relations within university structure (e.g., between professor and students), rather than to gender alone. While acknowledging that women are more frequently affected by SH, these accounts also highlighted that men can be victims, often based on their own experiences as bystanders.

*“Any behavior—regardless of gender—that, in an academic setting, involves an abuse of power.”* (UNIVR_11, SR, woman)

*“I think anyone, with any kind of characteristic, can experience sexual harassment. Here we don’t consider that some might be more vulnerable just because they are women and men are stronger, or because within women there are certain personalities or traits that make them more vulnerable to harassment.”* (UA_00, SR, woman)

*“It also happens a lot from woman to man, which I’ve witnessed at university.”* (UMAIA_10, SR, woman)

However, unlike the student body, staff and institutional representatives, especially women, justified this *situational discourse* with cautious language, avoiding reducing the phenomenon to a stereotypical victim profile or attributing blame to the victims. Participants´ narratives stemmed from their direct engagement in their professional position (e.g., legal advisors, psychologists, equality officers, equality representatives, supervising lecturers or tutors).

*“I don’t believe anyone is more or less at risk of sexual harassment […] There’s no specific cluster—it can happen to a student or to a 60-year-old woman alike.”* (UNIVR_06, IR, woman)

*“Let’s see, more than characteristics, maybe there are victims who are, let’s say, more vulnerable, because when we talk about profiles or characteristics, I don’t know, sometimes we can make the mistake of almost blaming the victims that they have certain characteristics [...] nobody is susceptible to being abused.”* (UA_10, US, woman)

1. **Representations about SH perpetrators**

Three discourses about SH perpetrators have been identified: hierarchical status discourse, peer perpetration discourse, and psychological characteristics discourse. The *hierarchical status discourse* emerged as the dominant perspective, emphasizing the role of power and authority in shaping perpetrator behavior. Alongside this dominant discourse, two alternative discourses were identified across the three interviewee profiles. The *peer perpetration discourse* highlighted incidents among students and staff of the same status. The *psychological characteristics discourse* focused on the individual traits and personality characteristics that participants associate with potential perpetrators.

***Hierarchical status discourse.*** Among students, staff, and institutional representatives, there was a clear discursive convergence about the potential profile of SH perpetrators. This dominant discourse, understood as recurrent and widely shared across participants’ accounts, characterized the perpetrator as a heterosexual man, usually older, with a sexist personality, who holds a position of responsibility or leadership (e.g., department coordinators or directors, tenured lecturers, thesis directors). This recurring profile reinforced the perception of power imbalances as a key element in situations of SH in the university context, where hierarchical status not only confers authority but also normalizes “untouchability”, favoring perpetrators to act with impunity.

*“The characteristics of the perpetrator…Generally, be a heterosexual man […] Fragile masculinity often tends to be a breeding ground for harassers.”* (UA_06, SR, woman)

*“They are either area coordinators or department directors [...]they are teachers who give classes to female students. Yes, it is usually the teacher who teaches them. And he acts a bit paternalistically.”* (UA_03, US, woman)

*“I recognize that older (male) teachers may still have some... temptation to think that it is an invitation to something more.”* (UMAIA_04, IR, woman)

*“If you are in a certain position, you can do whatever you want. Everyone can know that, but they [perpetrator] are not afraid”* (UAntwerp_10, US, woman)

*“His power is already so established that it becomes a normal thing.”* (UMAIA_06, SR woman)

*“Professors have a kind of untouchable status. [...] That is also why things like that happen and why people quickly say: ‹‹we can’t do much about it, because those positions are so untouchable››”* (UAntwerp_05, US, non-binary)

***Peer perpetration discourse.*** An alternative discourse, which had a limited presence across participants’ accounts, identified male students as perpetrators, mainly in cases of SH among peers, that is, perpetrated by male students toward female students. This perspective tended to explain SH as something rooted not only in power inequalities of university structure, but also in broader gender inequalities in society. More marginally, references also emerged to SH occurring among staff of the same hierarchical status, noting that such cases are generally easier to address because they do not involve relationships of formal dependency.

*“As I already emphasized, perpetrators are most often men, including young ones. I’m thinking of students. So it’s not always about professional or workplace subordination.”* (AMU_01, IR, man)

*“Teachers, also working people, but they [perpetrators]can also be students perfectly.”* (UA_15, SR, woman)

*“We had a number of, yes, male colleagues who were quite, how should I say this, a bit hormonally driven. And at the slightest, whenever someone [female] new came in, [they were] like, […] “pretty lady, we should go for a drink”.* (UAntwerp_12, US, woman)

*“It’s different when you receive certain kinds of comments from someone who is higher in the hierarchy than from someone at your own level; you can defend yourself more easily in the latter case, I would think.”* (UAntwerp_16, US, woman)

***Psychological characteristics.*** Another alternative discourse focused on individual and psychological factors attributed to the perpetrators. More specifically, some participants across the three interview profiles described the perpetrators as narcissistic, egocentric, manipulative, or even psychopathic. However, this subject position, was mainly present among staff and institutional representatives and coexisted with an ambivalence, where perpetrators were simultaneously portrayed as friendly, relaxed, and socially competent individuals who enjoy legitimacy and recognition within the academic community. This perpetrator’s image was often described as “easygoing-guy”, a brilliant professional, an exemplary student, or a supportive colleague/friend. This contradictory profile was further reinforced by accounts of perpetrators who publicly present themselves as feminist, egalitarian, or socially aware, which contributes to masking their behavior and complicates SH recognition by both victims and bystanders.

*“Others will certainly have psychopathic characteristics.”* (UMAIA_01, US, woman)

*“And also quite narcissistic people, right? Because they think they’re the center of the universe and that… people are going to say yes to them, yes or yes.”* (UA_13, SR, woman)

*“The kind of person who crosses boundaries – well, that’s a macho type, right? A macho guy with an inflated, oversized ego.* (AMU_04, IR, woman)

*“A sexist mentality and a sexist personality and a personality in which they consider themselves to be better than, of superiority.”* (UA_01, IR, woman)

*“He doesn’t have any of the characteristics that we often see or that are seen, [he was] someone with a completely excellent image.”* (UMAIA_03, US, woman)

*“In my opinion, it’s the one you’d least expect—the guy who seems easygoing, always joking around. […] He’s not some stereotypical monster, but an ordinary person who doesn’t show any obvious warning signs.”* (UNIVR_05, SR, man)

*“They’ll probably say, ´no, no, I support women… And I’m very feminist and for equality´.”* (UA_04, IR, woman)

Among female students, ambivalence emerged. Although they acknowledged that power imbalances are a key factor in SH cases within universities, they also frequently described perpetrators as acting without full awareness, attributing these inappropriate behaviors to the internalization and normalization of sexist cultural norms. Students mentioned that perpetrators sometimes act unconsciously, unaware of the consequences, or impulsively, without thinking before acting—especially in the context of online SH. Unlike students, female and male staff and institutional representatives generally acknowledged that perpetrators are fully aware of their actions and intentionally take advantage of their position to exert control.

*“The person displaying such behaviors may often do so unconsciously.”* (UMAIA_09, SR, woman)

*“They don’t think about the things they do; they just do them. Because I don’t think, in the case of sending a dick pic, that you sit down and think about it for a long time, you know? It’s just, ´boom´, and that’s it.”* (UA_13, SR, woman)

*“The perpetrator is the one in the position, apparently, of greater power and the victim of lesser power.”* (UMAIA_02, IR, man)

*“I understand sexual harassment as a type of behavior that mainly involves exploiting one’s advantage from a superior or hierarchical position to coerce or humiliate”* (AMU_04, US, woman)

## Discussion

The findings of this study show that key informants in the university community perceive SH as a complex and multidimensional phenomenon that encompasses a wide range of behaviors—physical, verbal, psychological, and online manifestations. Participants characterize potential victims as individuals occupying relational and structural positions of vulnerability, particularly young women at early stages of their academic or professional careers. In contrast, discourses identify men as the primary perpetrators, especially those holding hierarchically superior positions within the institution, such as lecturers or supervisors. However, discursive differences appear across participant profiles—shaped by their university position and gender—as well as ambivalences within their accounts, which reveal the complexity of the issue.

Participants construct the SH concept around four common key behavioral elements: the transgression of interpersonal boundaries, the absence of consent on the part of the recipient, the role of power imbalances, and the negative impact on victims’ well-being. Firstly, in line with previous literature [37], the transgression of interpersonal boundaries, manifested through intrusive behaviors such as physical contact, touching, gestures, unwanted comments, or messages, emerged as a central concern in the participants’ accounts. They described how these behaviors blur the boundaries between professional and personal interactions, creating inappropriate situations in which the targeted person may feel that their private sphere is violated or compromised. Online contexts and new technologies -such as smartphones and social media- seem to encourage this transgression of boundaries [48]. The examples cited by participants, such as receiving calls or messages outside of academic or labor timetables, highlight how online communication can extend interactions beyond the formal and academic relationship between students and lecturers. This is consistent with evidence suggesting a lack of knowledge or disrespectful attitudes among staff and students regarding the boundaries of professional interactions [48–50].

Secondly, the absence of consent and the notion of unwanted behaviours were consistently highlighted in participants´ SH definitions. According to their accounts, any interaction with sexual connotations that occurs without the target person´s consent is perceived as harassment, regardless of its apparent subtlety or ambiguity (e.g., unwanted comments or compliments about physical appearance or sexual jokes). This finding is in line with the classic SH definition proposed by Kast and Kleiner [7], which emphasizes the “communication by the victim that the behavior was unwelcome and the continuation of the disapproved behavior by the harasser, since this situation is usually not overt in nature” [7, p.158]. Thirdly, and closely related to the previous point, power imbalances emerge as a central explanatory factor for understanding SH in the university context. Hierarchies based on gender, age, academic level, and other interrelated inequalities create conditions in which unwanted sexual attention is more likely to occur [29,35,37]. In university settings, the experiences of SH survivors-particularly female students-are often shaped by these asymmetrical power relations, as faculty and other authority figures can significantly constrain their autonomy and ability to give, withhold, or withdraw sexual consent [37,49,51]. This illustrates how power dynamics can normalize and perpetuate SH among the university community.

As a last element, the participants- mostly women- highlighted the negative impact of SH on the well-being of those who experience it. Their accounts emphasized how these experiences have consequences across multiple dimensions of well-being, including emotional, physical, social, and academic functioning. A systematic review by Klein and Martin [52] showed that students who experience SH are more likely to suffer from psychological disorders, eating disorders, substance abuse problems, and lower academic performance. However, there are disparities in the severity of these consequences, as women and ethnic minority students report more severe psycho-emotional and academic consequences than men [30,36,52]. Research on the consequences of SH among university staff remains limited. Nevertheless, findings from the UniSAFE project [29] indicate that gender-based violence in universities and research institutions is associated with reduced work productivity, increased intentions to leave the academic career, lower engagement with colleagues, and decreased job satisfaction. These findings underscore the need for future research to take into account the role of social determinants—such as gender, age, position in the university, or ethnicity—in shaping the experience and consequences of SH in academia.

Participants’ definitions move along a continuum between normative, experience-based, and integrative dimensions, reflecting their recognition of the multiple and intersecting elements that constitute SH. Our findings reflect the historical ambiguity of SH in the literature between the normative perspective, which is based on law and institutional definitions, and the subjective perspective, which emphasizes recipient experiences, perceptions, and emotional responses [4–6,10]. The Critical Discourse Analysis used in this study further revealed different discursive positions aligned with participants’ institutional profiles and roles in the university. On the one hand, staff members tended to adopt a normative discourse emphasizing the importance of common frameworks (e.g., protocols, codes of conduct) for classifying SH. This position was often justified by reference to variability in individual boundaries, which, in their view, necessitates formal guidelines to ensure consistency in defining and identifying SH. Although staff recognize behaviors that cause discomfort or tension in the target person (e.g., persistent compliments or unwanted attention), they sometimes avoid labeling them as SH, describing them instead as “inappropriate persistence” or simply “flirting.” Consequently, this perspective highlights the challenges in addressing the gray areas of SH that are often normalized within formal institutional processes, where legal and regulatory criteria may overlook subtle or less visible behaviors [7,12,53]. In contrast, student representatives adopt a position closer to an experience-based discourse [37]. They recognized that SH includes behaviors extending beyond overt physical and visible forms, and emphasized the emotional impact of these behaviors on recipients (e.g., sexual jokes, unwanted compliments, sexually explicit song lyrics). Feelings of discomfort, stress, and tension were considered as key indicators in defining SH. This discourse may be explained by the fact that young people, especially female students, are more frequently exposed to inappropriate sexual behaviors in their everyday interactions [36], often occurring through online channels [54]. Institutional representatives tend to position themselves within an integrative discourse. This perspective represents a novel finding, as it seeks to balance the precision of regulatory frameworks with sensitivity to the perceptions, accounts, and experiences of victims-survivors. This integrative stance appears to constitute a relevant contribution of the present study, as it reflects the situated expertise of professionals directly involved in managing SH cases (e.g., equality officers, legal advisors, psychologists). Their accounts suggest that bridging formal regulation with experiential understanding may be important for strengthening institutional trust and encouraging reporting. Further research should continue to examine the perspectives of institutional actors involved in addressing SH and other forms of gender-based violence in academic settings.

Regarding perceptions of the potential profile of SH victims, the *intersectional discourse* characterized victims as individuals in situations of social vulnerability shaped by multiple intersecting social factors, including gender, age, early-career status, belonging to the LGBTQIA+ community or ethnic minorities, and people with disabilities. This finding confirms what has been noted by previous research in the field: SH is an expression of gender-based violence, and the risk of victimization is unequally distributed and influenced by individuals’ social circumstances [29,30,52,55]. This discourse was common and dominant across all profiles of key informants, particularly among women. However, an alternative perception, with a limited presence across participants’ accounts, also emerged: the *situational discourse*, which avoids identifying a specific victim profile based on sociodemographic characteristics. Instead, it emphasizes the subordinate position that victims tend to occupy within the university’s hierarchical structure, regardless of their gender or social background. From this perspective, the risk of SH victimization is not based solely on gender inequalities but is also influenced by other forms of power rooted in the academic structures, such as competitiveness and individualistic work cultures [29,55,56]. For instance, the academic dependence of students on lecturers or supervisors, as well as the employment and economic dependence of junior staff on senior academics, were highlighted as key conditions in SH victimization. Within this situational framework, some student representatives acknowledged that while women are more frequently affected by SH, men can also be victims, particularly those who do not conform to hegemonic ideals of masculinity [57]. Institutional staff and representatives tended to justify the “situational” discourse of SH as a way to avoid stereotyping victims and reducing the phenomenon to fixed or simplified profiles. In line with Humbert and Strid’s study [58], this perspective challenges the widespread conception that SH is exclusively a women’s problem. As these authors argue, reducing SH to a form of violence against women not only overlooks the experiences of men and non-binary individuals but also reinforces stereotypical assumptions about victimhood, often framed in terms of powerlessness. Although the two discourses identified in this study may appear contradictory, they converge in highlighting the role of power imbalances based on a combination of gender and other social factors within universities. Both discourses suggest the need to transform the dynamics of academic work towards equitable forms of organization, as well as integrating a social equity approach into future SH research, policies, and prevention strategies that explicitly address the structural power imbalances [41,42,59,60].

Three discourses around SH perpetrators emerged: hierarchical status discourse, peer perpetration discourse, and psychological characteristics discourse. The *hierarchical status discourse* emerged as a common and dominant discourse among three interviewee profiles, characterizing perpetrators as men in positions of authority, such as department coordinators or thesis advisors. This finding aligns with evidence that perpetrators often hold positions of power higher than those of their victims [57]. Notably, participants described how the intersection of formal authority and academic prestige may generate perceptions of “untouchability” surrounding powerful actors. Such perceptions may contribute to normalization processes and to a broader culture of silence within university environments. These dynamics resonate with the notion of “institutional network silence” [61], whereby organizational stability and reputation are prioritized over victim-survivor protection and accountability. By contrast, the *peer perpetration discourse* refers to SH incidents occurring between students or staff of the same hierarchical status. This discourse can be understood as a narrative that frames the SH perpetration through sexist behaviors and unequal interactions of men towards their female colleagues, rather than through formal authority structures. As an alternative discourse, participants in our study perceived peer SH as less frequent than hierarchical-based SH, yet generally easier to address due to the absence of academic or professional dependence. This finding contrasts with previous studies reporting a higher prevalence of peer-perpetrated SH compared to harassment by superiors [30,62]. However, it is important to note that much of the existing literature is limited to studying peer SH among students. Future research should therefore extend the analysis of peer-perpetration SH among staff, considering how work dynamics, informal relationships, and gender norms shape SH.

Finally, although interviewees mainly referred to social factors associated with perpetration, the *psychological characteristics discourse* also emerged as an alternative position, especially among staff and institutional representatives, highlighting individual traits of perpetrators such as egocentrism and manipulation. Along the same lines, the study by Arief et al. [63] demonstrates a strong association between maladaptive personality traits and the SV perpetration. Nevertheless, research focusing specifically on SH and SV perpetrators in university settings is scarce [55]. Future university policies and interventions should be primarily informed by the voices and experiences of victims-survivors, while also taking into account the perspectives of perpetrators, bystanders, and the broader university community.

This study includes some limitations that should be considered. First, participants may not have directly experienced or witnessed SH incidents, but the analysis focused on their discourses as key informants rather than on first-hand accounts. Moreover, participants’ roles as university representatives may have increased their awareness of and sensitivity to SH, potentially shaping their discourses. Nevertheless, this also implies that their accounts reflect a situated perspective grounded in institutional experience, which may not fully capture the experiences of other university members. Third, although we aimed to reach a diverse sample in terms of gender, occupational and academic profile, and membership in social organizations, the sample was predominantly female (68 women, 21 men, 1 non-binary), which may have limited the diversity of discourses and made it difficult to explore potential gender-related differences. In addition, given the sensitive nature of the topic, responses may have been influenced by social desirability bias, as interviewees might have felt the need to align their accounts with socially acceptable or institutionally appropriate positions. To mitigate this risk, interviewers fostered open dialogue and ensured anonymity and confidentiality throughout the research process. Finally, the cross-cultural design of the study, involving participants from different university and national contexts, constitutes both a strength and a limitation. While it enabled the identification of shared discursive patterns across contexts, differences in legal frameworks, institutional policies, and sociocultural understandings of SH may have shaped participants’ perceptions and interpretations. Consequently, the findings should be understood as reflecting discourses constructed within diverse cultural and institutional settings rather than as directly comparable across contexts.

## Conclusions

This study provides insight into how members of the university community define and perceive SH, which in turn can influence their attitudes and responses. Participants conceptualize SH as a continuum encompassing normative, experience-based, and integrative dimensions, including physical, psychological, verbal, non-verbal, and online forms. Discursive responses vary according to gender and university position: female students tend to be more sensitive to subtle forms of violence, drawing on their experiences as victims or bystanders and emphasizing experience-based perspectives aligned with survivor narratives; staff representatives often adopt a more normative perspective, focusing on regulatory frameworks and institutional roles to address diverse individual sensitivities and the gray areas of SH. Both male and female institutional representatives seek to balance experience-based and normative approaches, guided by their professional responsibilities in managing SH cases. Ambivalences emerged throughout the data, highlighting the complexity, contextuality, and multidimensionality in understanding and addressing SH. Power imbalances and hierarchical structure were identified as key elements of SH. Although women in particular emerged as a potential victim profile, other social determinants also were perceived as relevant, including students and employees in lower positions, LGBTQIA+ people, ethnic minorities, and people with disabilities. Similarly, perpetrators were predominantly perceived as men occupying positions of superiority over women and other marginalized groups, reflecting the structural and relational nature of power within academic settings. These findings underscore that the dynamics of SH cannot be understood in isolation from broader social hierarchies and institutional contexts. Our evidence suggests that preventing SH in universities requires integrating a gender-transformative and intersectional approach into institutional policies, fostering university-wide awareness and a culture of zero tolerance, and promoting healthy, egalitarian relationships and work dynamics. More specifically, prevention efforts should prioritize SH training that reaches the entire university community, particularly groups that face heightened vulnerability. These initiatives should explicitly address the role of power imbalances and hierarchical transgression in shaping vulnerability to SH and other forms of gender-based violence in academia. At the same time, it is necessary to develop and implement clear codes of conduct and equality plans that define unacceptable behaviors, including abuse of power, and establish accountability procedures. Strong institutional frameworks should be complemented by prevention and response strategies that are grounded in the voices and lived experiences of victims and survivors.
